# Promoting research and audit at medical school: evaluating the educational impact of participation in a student-led national collaborative study

**DOI:** 10.1186/s12909-015-0326-1

**Published:** 2015-03-13

**Authors:** Stephen J Chapman, James C D Glasbey, Chetan Khatri, Michael Kelly, Dmitri Nepogodiev, Aneel Bhangu, J Edward F Fitzgerald

**Affiliations:** 1 University of Leeds, Leeds, UK; 2 University of Cardiff Medical School, Cardiff, UK; 3 Imperial Medical School, London, UK; 4 University of Liverpool, Liverpool, UK; 5 University of Birmingham, West Midlands, UK; 6 West Midlands Research Collaborative, West Midlands, UK; 7 Division of Medical Education, UCL Medical School, University College London, Gower Street, London, WC1E 6BT UK

**Keywords:** Surgery, Training, Research, Audit, Academia

## Abstract

**Background:**

Medical students often struggle to engage in extra-curricular research and audit. The Student Audit and Research in Surgery (STARSurg) network is a novel student-led, national research collaborative. Student collaborators contribute data to national, clinical studies while gaining an understanding of audit and research methodology and ethical principles. This study aimed to evaluate the educational impact of participation.

**Methods:**

Participation in the national, clinical project was supported with training interventions, including an academic training day, an online e-learning module, weekly discussion forums and YouTube® educational videos. A non-mandatory, online questionnaire assessed collaborators’ self-reported confidence in performing key academic skills and their perceptions of audit and research prior to and following participation.

**Results:**

The group completed its first national clinical study (“STARSurgUK”) with 273 student collaborators across 109 hospital centres. Ninety-seven paired pre- and post-study participation responses (35.5%) were received (male = 51.5%; median age = 23). Participation led to increased confidence in key academic domains including: communication with local research governance bodies (p < 0.001), approaching clinical staff to initiate local collaboration (p < 0.001), data collection in a clinical setting (p < 0.001) and presentation of scientific results (p < 0.013). Collaborators also reported an increased appreciation of research, audit and study design (p < 0.001).

**Conclusions:**

Engagement with the STARSurg network empowered students to participate in a national clinical study, which increased their confidence and appreciation of academic principles and skills. Encouraging active participation in collaborative, student-led, national studies offers a novel approach for delivering essential academic training.

**Electronic supplementary material:**

The online version of this article (doi:10.1186/s12909-015-0326-1) contains supplementary material, which is available to authorized users.

## Background

The appreciation and application of research and audit principles is widely recognised as a core component of all medical school curricula [1]. In the United Kingdom (UK), medical school curriculum guidance is set out by the General Medical Council’s (GMC) ‘Tomorrow’s Doctors’ (2009) [2], which calls for proficiency in evidence-based medicine, formulation of research questions and an understanding of ethical governance (Table 1). Such expectations are similar in other European countries such as Germany, where a period of formal undergraduate research and resulting thesis submission is mandatory prior to assuming the title of ‘Doctor’ [3]. However, numerous practical, cultural and political barriers restrict medical students’ participation in high quality audit and research projects, including a perceived lack of time and opportunity, hostile environments and inadequate academic training in medical school [4-7].

**Table 1:** **Research related outcomes for graduates,** ***Tomorrow Doctors 2009*** **(General Medical Council, 2009)**

Item:	Apply scientific method and approaches to medical research:
(a)	Critically appraise the results of relevant diagnostic, prognostic and treatment trials and other qualitative and quantitative studies as reported in the medical and scientific literature
(b)	Formulate simple relevant research questions in biomedical science, psychosocial science or population science, and design appropriate studies or experiments to address the questions.
(c)	Apply findings from the literature to answer questions raised by specific clinical problems.
(d)	Understand the ethical and governance issues involved in medical research.

Medical student participation in audit and research offers a practical opportunity to explore the application of evidenced-based medicine [8]. It provides a unique and intellectually challenging environment for self-development, which may enrich students’ clinical capabilities as medical graduates [9]. Early exposure to research may prompt interest in academic pursuits [10,11], which in turn may address a potential shortfall in academic faculty in the future [12,13].

Trainee-led research collaborative networks offer a novel and innovative approach to undertaking high quality, multicentre clinical research projects and are gaining popularity [14]. Through the combined efforts of nationally placed collaborators, studies are delivered with larger populations, improving the external validity of results, and within a shorter timeframe. A number of recent high-impact clinical studies, such as the ROSSINI trial and National Appendicectomy Audit, have successfully utilised this approach [15,16]. Medical students and junior doctors are ideally placed to contribute to such large, national research and audit projects as they form a natural network across all medical schools and teaching hospitals.

Inspired by the trainee collaborative research model, a national medical student-led network has been established with representation from all UK medical schools: the Student Audit and Research in Surgery (STARSurg) group. This collaborative network empowers students to participate in high quality academic projects, forming links with supervising junior doctors and consultants. Through this, students contribute data to national studies while gaining an understanding of clinical academia, audit and research methodology, and ethical considerations.

This study aimed to evaluate changes in medical students’ confidence and perceptions of clinical research and audit through participation in the STARSurg annual, national study.

## Methods

### Setting

There are currently 33 UK undergraduate medical schools recognised by the Medical Schools Council [17]. A recent cross-sectional study of 34,407 UK medical students described demographics of 60% female, 71% white ethnicity and 39% from higher professional backgrounds [18]. Enrolment is competitive and varies between institutions with a mixture of pre-medical, undergraduate and postgraduate courses. The content of UK medical curricula and their teaching strategies vary between institutions, although all are approved and regulated by the GMC. As such, formal teaching and exposure to academic principles and skills is variable. In the UK, medical degrees are typically 5-year courses for first-degree undergraduate entry, extending in duration in institutions where research-based intercalated degrees are offered. At least one third of students undertake an intercalated degree, although this proportion is rising as medical schools are increasingly integrating these degrees into their curricula [19]. Graduate entry medicine courses for students having already completed their first degree are typically 4-years in duration.

### STARSurg collaborative network

**Figure 1:**
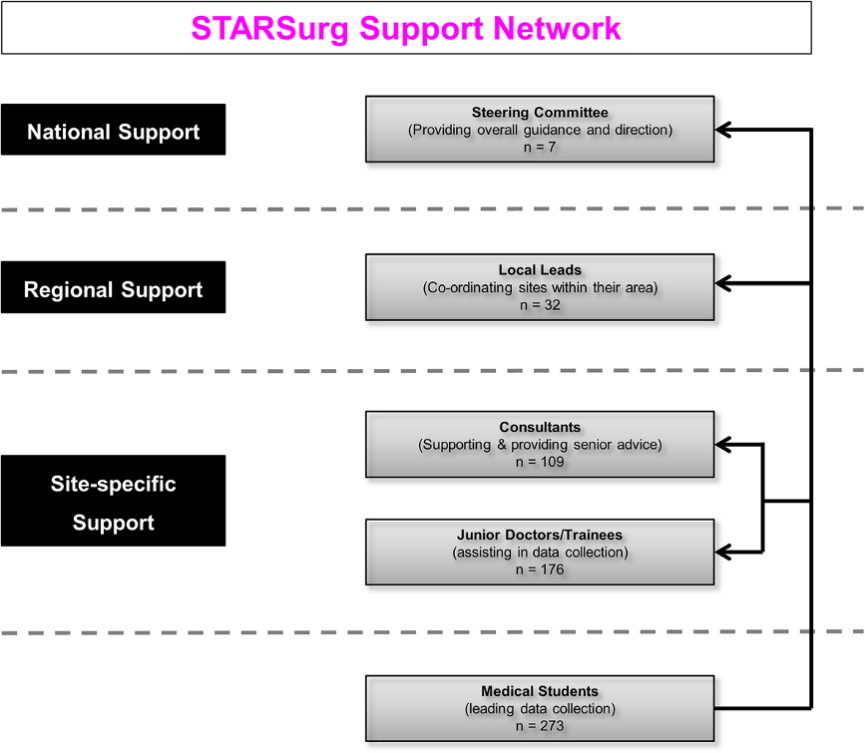
**Structure of STARSurg Mini-team support model.**

**Figure 2:**
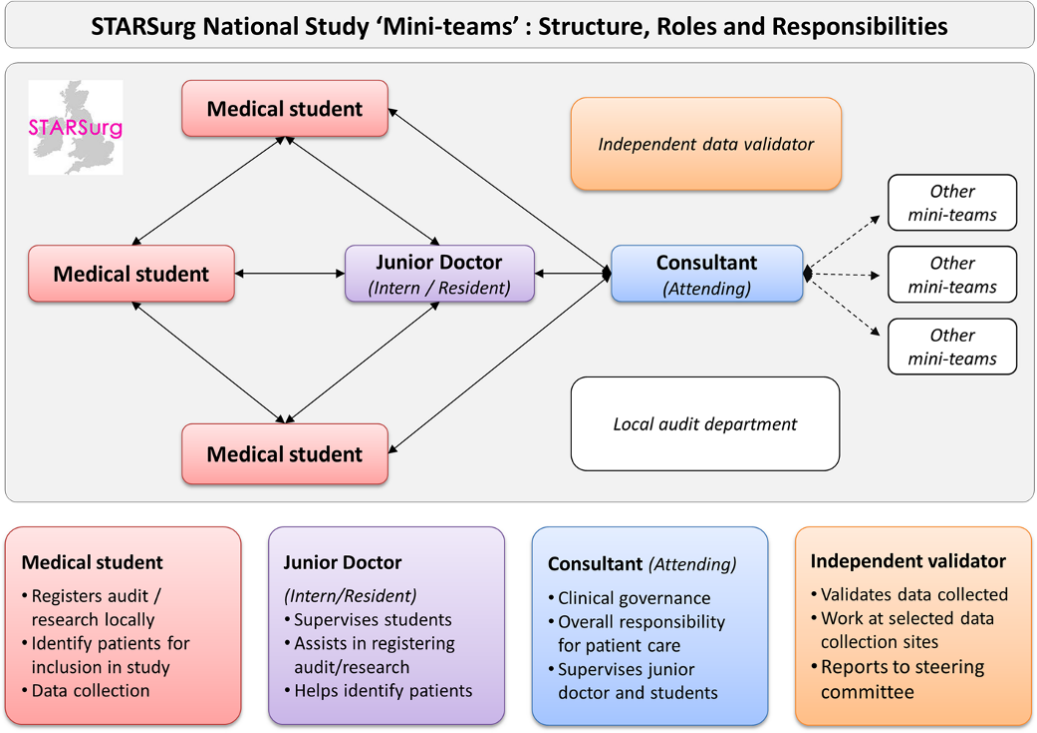
**Structure of the STARSurg collaborator model.**

The STARSurg Collaborative Network aims to foster academic potential in medical students by offering a novel, extra-curricular experience of academic research and audit. All UK medical students were eligible to join the network, which was promoted through local and national medical school mailing lists and social media. Students were recruited and organised locally into “mini-teams” by regional student coordinators and allocated to specific hospital units for data collection. Mini-teams are small groups of students with a linked junior clinician. They act as data collectors, who then feedback to a local lead and submit anonymised data centrally to the steering team, who analyse the findings. Collaborators were supervised within a structured hierarchy of support, comprising local clinical faculty and a dedicated steering committee, including senior clinicians with experience in large-scale clinical research (Figure 1). The overall structure of the STARSurg collaborator model is illustrated by Figure 2.

### STARSurg national study

The first STARSurg study (“STARSurgUK”) was delivered using a protocol-driven, student-led approach. The clinical aim of STARSurgUK was to determine the general safety profile of non-steroidal anti-inflammatory drugs (NSAIDs) following gastrointestinal (GI) surgery, in light of evidence suggesting a greater risk of post-operative anastomotic leak when administered peri-operatively [20,21]. In contrast, the results of the STARSurg study suggested no such risk exists, and in carefully selected patients the risk of anastomotic leak is reduced with administration of NSAIDs [22]. The paper was published with full collaborative authorship and will fuel further international debate on this important issue in the GI surgery community.

The educational aim of STARSurgUK was to promote the principles of evidence-based audit and research amongst UK medical students. Dedicated training initiatives were designed to complement the practical experience received through participation in the study by instilling essential academic skills and principles. They also served as a quality control measure for the clinical study and assisted in providing internal project evaluation.

Training initiatives included:

1. *Academic Research Training Day*: All collaborators were invited to a dedicated training day at the Royal College of Surgeons of England (London), which sought to increase awareness of general academic principles and specific issues surrounding the STARSurgUK study protocol. The day provided a forum for discussion prior to commencing the clinical study. Participation was optional.
2. *Clinical Outcome E-Learning Module*: This aimed to instil a clear understanding of the primary outcome measure for the clinical study (Clavein-Dindo Classification system [23] – an outcome measure students may not be routinely familiar with) and was delivered via a free web-based platform (QuizStar®, http://quizstar.4teachers.org/) [24]. Collaborators were required to successfully complete an assessment to validate their understanding, acting as an important quality control measure for participation in the clinical study. This comprised a series of clinical scenarios which collaborators scored using the Clavien-Dindo criteria. The pass mark was 100% and successful completion prior to participation in the study was mandatory. The module was undertaken in the participants’ own time from any convenient online-accessible computer.
3. *Weekly Live Twitter® Forum*: Twitter® is a popular, online, social media micro-blogging service. A weekly, moderated forum for peer discussion of the STARSurgUK study and general academic issues was held during the six-week study period. The forum was hosted via the @STARSurgUK Twitter account (https://twitter.com/STARSurgUK) using the #STARSurg hash tag. Participation was optional.
4. *Pre- and Post-Study Debrief Videos:* Online video learning resources were developed before and after the clinical study to explore the study aims and objectives and to later reflect on progress and areas for self-development. These were hosted on YouTube® (http://www.youtube.com), a popular, online video sharing platform. Viewing was optional.

**Figure 3:**
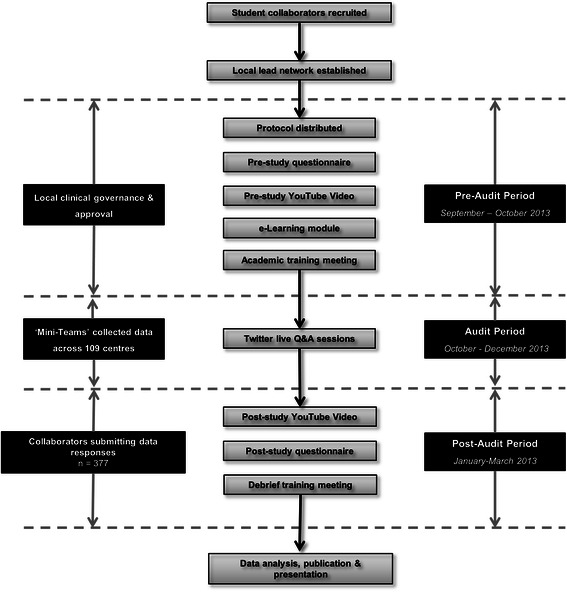
**Flowchart detailing study stages including educational program.**

Figure 3 provides a flowchart detailing the training initiatives and their timing within the study.

### Questionnaire development

A 27-item pre- and post-study questionnaire was developed with reference to previously published guidelines on questionnaire research [25,26]. This sought to investigate students’ perceptions and opinions of audit and research, their self-reported confidence in performing core academic skills and their future career aspirations. The primary outcome measure was the relative change in self-perceived confidence in key academic domains following involvement in the clinical study.

The questionnaire was hosted via a dedicated online-based platform (Survey Monkey®, Menlo Park, CA [surveymonkey.com]), and comprised of five-point Likert scales and free text response questions as appropriate. Answer randomisation was enabled and a mixture of positively- and negatively-framed questions were used to minimise response bias. The questionnaires were piloted on senior medical students and junior doctors to ensure content and face validity. Written feedback was received and used to iteratively refine the question items to improve clarity and understanding. Responses to the pre- and post- questionnaire were paired by email address, which was disclosed by participants. A complete copy of the pre- and post-participation evaluation questionnaires are included as Additional files 1 and 2.

### Questionnaire distribution

All student collaborators were invited to participate in the online-based questionnaires over a 5-month period, with invitations sent via email to their registered personal email address. The pre-study questionnaire was distributed prior to the start of the clinical study and the post-questionnaire was distributed during a four-week period immediately following study completion. Participation was non-mandatory and did not attract incentives or benefits. Internet Protocol (IP) address blocking was activated to limit responses to one per invitation. Reminders were sent at regular intervals to maximise the response rate; non-responders were emailed individually with a final invitation.

### Ethical approval and governance

The clinical study protocol was reviewed by the National Research Ethics Committee and deemed exempt from full ethical consideration. As an evaluation of participant opinions, ethical approval to conduct the questionnaire survey was not required, however appropriate ethical principles were considered at all stages. The participants’ medical schools were not identified. Email addresses were collected in order to match pre- and post-participation responses and were then removed prior to anonymous collation and analysis. The questionnaire was optional, and not required for participation in the prospective clinical study. Completion was taken as consent to participate.

### Statistical analysis

Only paired pre- and post-questionnaire responses were included in the analysis. Descriptive data were analysed using percentages (%) and medians. Paired responses were analysed using the Wilcoxon Rank Sum Test for non-parametric data. The relationship between number of patients audited during the clinical study and change in confidence scales was explored using Spearman’s Rho Correlation Coefficient. Differences were considered statistically significant if p < 0.05 was achieved. Statistical analysis was undertaken using the Statistical Package for the Social Sciences, SPSS 20.0 for Windows (SPSS Inc; Chicago, Illinois). Survey sample size and confidence interval calculations were based on standard published formulae [27].

## Results

### Demographics

A total of 273 medical students from 32 UK medical schools participated and collected data in 109 hospitals across the UK. Some 151/273 (55.3%) pre-study and 121/273 (44.3%) post-study responses were received from the total population. This yielded a response of 97/273 (35.5%) paired questionnaires with demographics outlined in Table 2. Similar numbers of male (n = 50/97, 51.5%) and female (n = 47/97, 48.5%) collaborators responded to both questionnaires, with a median age of 23 (range 19–35). All student seniorities were represented, although 95/97 (97.9%) of responses received were from students in senior clinical years of study (years 3–7).

**Table 2:** **Participant demographics (n = 97)**

Demographic		n=	%
Gender	Male	50	51.5
	Female	47	48.5
Age	18-21	21	21.6
	22-25	65	67.0
	>25	11	11.3
Medical School Year	1-2	2	2.1
	3-4	59	60.8
	5-7	36	37.1
Previous Degree	Yes – intercalated	32	33.0
	Yes – non-intercalated	21	21.0
	No	44	45.4
Previous Collaborative Research	Yes	2	2.1
	No, but aware of opportunity	44	45.4
	No, not aware of opportunity	51	52.6
Current Career Aspirations	Surgical Disciplines	65	67
	Other Disciplines	32	33
Previous Academic Output*	Yes	43	44.3
	No	54	55.7

*A composite measure of previous peer-reviewed PubMed-indexed publication or abstract presentation at a professional academic conference.

Most collaborators disclosed no previous engagement with research collaborative networks (n = 95/97, 97.9%), and many were unaware of their existence (n = 51/97, 52.6%). A substantial number of students (n = 43/97, 44.3%) described previous academic outputs, including conference presentations (n = 40/97, 41.2%) and peer-reviewed publications (n = 12/97, 12.4%). Of these students, the median number of presentations and publications was 2 (range: 0–5) and 0 (range: 0–3) respectively. Despite the focus of STARSurg on surgery, the study attracted students interested in a range of surgical (n = 65/97, 67.0%) and medical specialties (n = 32/97, 33.0%). Of those interested in other specialties, the five most popular areas of interest included emergency medicine (n = 11/32, 34.4%), general medicine (including all sub-specialties) (n = 5/32, 15.6%), obstetrics & gynaecology (n = 3/32, 9.4%), ophthalmology (n = 3/32, 9.4%) and paediatrics (3/32, 9.4%). Interest in general practice was relatively low in this cohort (n = 2/32, 6.3%).

### Sample size and confidence interval

A post hoc two-tail test calculation was undertaken to assess the power of detecting significant changes in Likert scale responses. With 97 paired responders, the study achieved >90% power (alpha 0.05) to detect a 1-point scale change based on the sample’s standard deviation of +/− 1. Given the population size and number of respondents, the margin of error was calculated as 6.6% at a 90% confidence interval.

### Participation in study training initiatives

Table 3 describes the level of participation in each training initiative which was integrated into the STARSurgUK study. A total of 86/97 (88.7%) collaborators participated in at least one optional training initiative. Respondents engaged most frequently with the YouTube debrief videos (n = 80/97, 82.5%); fewer attended the National Collaborators’ Meeting (n = 32/97, 33.0%) or participated in the live Twitter® forum (n = 26/97, 26.8%).

**Table 3:** **Respondent uptake of STARSurg training initiatives (n = 97)**

Training initiative		n=	%
Primary outcome measure E-module (Mandatory)	Yes	97	100
	No	0	0
YouTube® pre-study presentation (Optional)	Yes	66	68.0
	No	31	32.0
YouTube® post-study presentation (Optional)	Yes	65	67.0
	No	32	33.0
National collaborators’ meeting (Optional)	Yes	32	33.0
	No	65	67.0
Weekly Twitter® forum (Optional)	Yes	26	26.8
	No	71	73.2
YouTube® pre/post-study presentation composite*	Yes	80	82.5
	No	17	17.5
Optional training initiative composite^†^	Yes	86	88.7
	No	11	11.3

*Composite uptake of pre- or post-Youtube® videos.

^†^Composite uptake of at least one training intervention.

### Confidence in core academic competences

Student collaborators reported increased self-rated confidence in a range of key academic competences after participation in the STARSurgUK study (Table 4). The median number of patients audited per hospital centre was 11. Greater appreciation of audit and research practice (p < 0.001) and the quality improvement cycle (p < 0.001) were notable findings. Collaborators also felt more confident in their ability to construct a study protocol (p < 0.001), collaborate locally to establish a research or audit project (p < 0.001), work as part of a local clinical team (p < 0.001), engage with local study approval processes (p < 0.001) and collect data from clinical case notes and hospital computer systems (p < 0.001). In addition, collaborators felt more confident in disseminating their findings to local clinical faculty (p = 0.004). There was no significant relationship between number of patients audited during the clinical study and relative change in confidence across all domains.

**Table 4:** **Confidence in key academic domains before and after STARSurg engagement**

How confident do you feel in the following research domains? (1 = Very unconfident; 5 = Very Confident):	Pre-study (mean ± SD)	Post-study (mean ± SD)	*p=
Distinguishing the differences between audit, service evaluation and research	3.17 ± 0.92	3.81 ± 0.82	<0.001
Knowledge of the clinical audit cycle	3.29 ± 1.00	3.97 ± 0.68	<0.001
Writing an audit or research protocol	2.64 ± 0.90	3.38 ± 0.81	<0.001
Approaching clinical staff to help you formulate an audit/research protocol	3.24 ± 1.01	4.03 ± 0.65	<0.001
Approaching clinical staff to form a team to help you complete an audit/research protocol	3.4 ± 1.0	4.24 ± 0.58	<0.001
How to fill out an audit registration form	2.88 ± 1.05	4.10 ± 0.83	<0.001
How to contact your hospital’s clinical audit department	3.17 ± 1.03	4.15 ± 0.83	<0.001
How to collect data in the clinical setting	3.50 ± 0.89	4.43 ± 0.52	<0.001
How to present your results in a scientific manner	3.23 ± 1.13	3.65 ± 0.79	0.004

*Wilcoxon signed rank test for non-parametric data. p < 0.05 statistically significant; SD = standard deviation.

### Attitudes to research and audit

Attitudes towards the importance of audit and research as a medical student were positive but remained unchanged (p = 0.116) following participation. Students felt that access to academic opportunities was more straight forward (p = 0.004) following study completion (Table 5). Collaborators’ awareness of academic training pathways showed a positive trend, although this was non-significant (p = 0.157). No difference was observed in the proportion of collaborators intending to pursue an academic career in the future (p = 0.113).

**Table 5:** **Attitudes to research and audit before and after STARSurg engagement**

Please indicate your agreement with the following statements (1 = Strongly Disagree; 5 = Strongly Agree*):	Pre-study (mean ± SD)	Post-study (mean ± SD)	^ † ^ p=
Participation in clinical audit IS straightforward	3.40 ± 0.78	3.72 ± 0.65	0.004
Participation in audit IS important and relevant as a medical student	4.29 ± 0.67	4.44 ± 0.58	0.116
I AM aware of the structure of academic training pathways in the UK	3.65 ± 1.01	3.86 ± 0.80	0.157
I am NOT interested in pursuing a career in clinical academia*	2.98 ± 1.13	3.30 ± 1.12	0.113
I would NOT be interested in participating in a trainee -led research collaborative project in the future*	4.41 ± 0.75	4.35 ± 0.84	0.425

*Reverse framed question (1 = Strongly Agree; 5 = Strongly Disagree).

^†^Wilcoxon signed rank test for non-parametric data. p < 0.05 statistically significant.

SD = standard deviation.

### Perceptions of collaborative research opportunities

The majority of collaborators (n = 61/97, 62.9%) disagreed or strongly disagreed that collaborative research opportunities between medical schools were easily accessible. The majority (n = 67/97, 69.1%) agreed or strongly agreed that engagement with the STARSurg collaborative network had made it easier to participate in such activities. The number of collaborators calling for greater inter-school, surgically-themed networking opportunities was high before (n = 79/97, 81.4%) and after (n = 83/97, 85.6%) the study. Although 95/97 collaborators (98.0%) had no previous engagement with trainee-collaborative networks prior to the STARSurgUK study, 90/97 (92.8%) and 97/97 (100.0%) expressed an interest in future engagement with trainee collaborative networks and STARSurg respectively.

## Discussion

Participation in this medical student-led collaborative research and audit network is a novel medium for engaging students in academic projects. Our results indicate that through facilitating supervised, focused engagement in a high quality study, student collaborators can gain significant improvements in confidence within a number of core academic domains. Although improvements in professionalism and team working skills were not formally assessed, the nature of this collaborative study served as an ideal platform to develop these.

This project was designed to overcome previously reported barriers to student engagement in extracurricular academic projects [5,6]. By providing a detailed protocol with outcomes carefully selected to be achievable by medical students, participants were empowered to engage with junior and senior clinicians to conduct the clinical study. The educational package developed to support this process gave specific attention to the necessary practical learning outcomes required by students, such as how to obtain local audit registration through to identifying the necessary data collection points required and where to find these. More broadly, the novel “mini-team” local peer support structure provided on-going information and encouragement whilst stimulating broader discussion of generic academic principles and learning objectives.

Medical student involvement in research is a longstanding tradition. Historical examples where medical students have had a significant role include the discovery of Islets of Langerhans, heparin, the sino-atrial node and ether anaesthetic [28,29]. In modern times, the importance of understanding key academic principles during undergraduate medical training is upheld in the UK by the GMC. In their publication *Tomorrow’s Doctors 2009* [2], clinicians are described according to their role as “*scientists, practitioners* and *professionals”.* As future doctors, medical students must achieve outcomes related to each of these roles and be able to integrate the principles into clinical practice and their relationships with colleagues and patients. Countries outside of the UK adopt a similar outlook on student research; in Germany, for instance, student participation in research is mandatory for completion of their primary medical degree [3].

However, the number of clinical academics in the UK is declining. It is now 10% lower than in the year 2000 [12], with numbers of dedicated academic posts falling year-on-year [13]. It is therefore important that efforts are made to foster a new generation of enthusiastic clinician-scientists to combat this negative trend. Various UK specialty colleges and postgraduate institutes have already made attempts to create a more cohesive working environment for student researchers. Notable examples include the *Academy of Medical Sciences’* ‘INSPIRE’ initiative [30] and the *Institute for Health Improvement’s* ‘Open School’ [31].

A number of studies have explored the nature of student involvement in academic research, the motives behind this, and how these have changed over time. Recent surveys from the UK and overseas have demonstrated positive student perceptions of academia, with between 85% and 97% of students regarding research as an important undertaking respectively [5,6]. A number of other studies have indicated a strong desire to participate in research projects by students and a motivation to achieve peer-reviewed publications [32,33]. The enthusiasm of medical students to engage in research is particularly evident from the growth of student-authored peer-reviewed publications over previous decades. A recent cross-sectional assessment of PubMed-indexed publications [8] highlighted an exponential growth in student-authored papers, which is at least in line with the overall growth seen in medical literature over the same period [34]. Furthermore, medical school programme managers are increasingly recognising the importance of early exposure to academia, with reports of integrated curricular research training programmes, met with positive attitudes by students [35,36].

The motivation behind such involvement however, has been debated. Such interest in academic activities may be due to increasing availability of student-targeted programmes and other means of financial support [37,38]. Individual motivations may also relate to Curriculum Vitae-focused development, particularly in the UK where career progression is highly competitive and criteria-driven [5]. Importantly, this increasing level of engagement has taken place in the presence of continued barriers to student-research. Such barriers are well reported and include unfavourable or hostile environments, lack of formal supervision, training courses and funding [5,6]. On one hand this highlights students’ perseverance to engage in academic research, but also identifies areas of further work to overcome these continued barriers.

In the present study, it was evident that medical students were not completely research-naïve, with 44.3% reporting previous engagement in projects leading to notable academic outputs. While one international study has previously reported broadly similar levels of research engagement [7], these figures contrast with lower rates reported across one UK medical school [5], where only 38% final-year students described previous research participation, with fewer than three quarters achieving any academic output. One explanation for this may be the aforementioned general growth in academic activity in recent years. Alternatively, it may suggest a degree of bias towards those already possessing research or audit experience, through self-selection of study participants. This is suggested by an apparent disparity in participant demographics relative to those seen nationally, with a greater number of males, individuals with previous research exposure and degrees [39]. Although the impact of this is difficult to quantify, one study has previously suggested that graduate-entry medical students perform significantly better in research-based assessments than their undergraduate colleagues [40]. Nearly a quarter of participants in this study had previously received a non-intercalated degree (Table 2), which may suggest that the study population is skewed towards individuals who already hold an interest in academia, or at least a desire to explore it further.

Despite this finding, many students in the present study reported significantly increased levels of confidence and appreciation of key academic principles. One possible explanation for such broad improvements is the tendency for students to participate in audit and research projects which are not accompanied by formal training. Although this may be beneficial for short-term portfolio development and career progression, students may miss training in important underlying principles. In addition, the problem may be augmented by variable provision in formal research and audit training in UK medical schools. Although research charities offer opportunities such as student vacationships, which may involve an element of training, these are competitive, limited in number and available only during vacation periods, which may be unfavourable.

In contrast, the results of the present study demonstrated that participation had little impact on student attitudes to academic career aspirations, with respondents remaining neutral with respect to this. This is an important consideration owing to the decline in numbers of UK clinical academics over recent years [41]. Many strategies are being implemented in an attempt to reverse this trend, primarily by optimising postgraduate academic training opportunities [31,32]. Additional factors may influence or discourage interest in academic career pathways, such as competition for posts, time out of clinical training, desire to teach and financial rewards or sacrifices [42]. While focussed participation in this project has positively influenced self-reported knowledge of several key academic domains, this alone may not be sufficient to increase interest in academic careers more widely without these broader factors being addressed.

Overall, these findings suggest that students recognise academic research as an important component of medical training, regardless of their interests and future career intentions. Indeed, STARSurg is primarily focussed on surgical academic projects, yet students with a broad range of medical and surgical interests participated in the study, suggesting that core principles rather than context was a major determinant in participation. Interestingly, participation in the initiative did not lead to significant advances in appreciating the role of audit in clinical practice. As discussed previously, this may be due to disparity in students’ agenda for participation, with audit seen as a requirement for career progression rather than a quality improvement process. Alternatively, this may be explained by current limitations of the STARSurg model, which does not mandate national re-audit, but strongly encourages local closure of the audit cycle.

The majority of students were unaware of the existence and activities of post-graduate trainee collaborative research and audit groups, with only two students describing previous exposure. Although the model of trainee-led collaborative research groups is relatively new [15], this finding suggests that more needs to be done by medical schools and collaborative groups to raise awareness of such opportunities to local medical students. This in turn may introduce further long-term benefits for students. The network of support created by the clinical study may promote further collaborations and additional opportunities to engage in academic activities with senior clinicians and fellow students [43]. Previous evidence has identified greater academic output by clinicians who gained extracurricular experience of projects at medical school [10,11]. Therefore, engagement in organised and supported networks may help to promote engagement in academic research in the future.

A wide national scope was a significant strength of this study, with student participation from 32/33 (97%) medical schools. Given the likely variation in academic research experiences between students at different medical schools, this wide participation gives support to the broad applicability of the findings. In addition the longitudinal design of this study permitted an assessment of outcome effect, rather than being limited to a simple description of outcomes.

Further limitations in this study are acknowledged. Online surveys inherently yield low response rates, often achieving only 25-30% of the target population [44]. During the present study, reminder emails were sent to all non-responders according to a dedicated protocol, which achieved high response rates of 55.5% and 45.5% for the pre- and post-questionnaire respectively. However, owing to the longitudinal nature of this study, only paired pre- and post- responses were analysed, yielding a lower final response rate. Importantly, students self-selected themselves to participate in this national project, which may bias the findings towards those with higher pre-existing levels of interest in research and audit projects. Although positive outcomes are reported, it is unclear to what extent these could be extrapolated to the wider medical school population.

Finally, it is important to note that this study reports the impact of our educational intervention only at the reaction level. Longitudinal studies may help explore the impact of such standardised initiatives on future participation in research and uptake of academic careers, or other objective outcomes of academic engagement and impact. Future work may also focus on the role of integrating other promotional initiatives into medical school curricula, in addition to existing, medical school-specific provisions to encourage academic engagement.

## Conclusion

This novel, collaborative study provided medical students with a unique, applied academic training experience. Collaborators reported significantly increased appreciation and confidence in relation to core academic principles. Although participation did not impact on students’ intentions to engage in academic careers, engagement may serve as a valuable adjunct to instilling essential academic skills. We recommend and encourage student participation in high quality, collaborative, student-led, supervised projects to deliver extracurricular academic training.

## Additional files

Additional file 1:
**Pre-questionnaire.**
Additional file 2:
**Post-questionnaire.**
