# Supplementary material for: Promoting research and audit at medical school: evaluating the educational impact of participation in a student-led national collaborative study
Source: BMC Med Educ. 2015 Mar 13;15:47. doi: 10.1186/s12909-015-0326-1 (PMC4456723; doi:10.1186/s12909-015-0326-1)
Supplement: Additional file 1: — Pre-questionnaire. [file 12909_2015_326_MOESM1_ESM.pdf]

Student Experience Survey (Pre)

Introduction to STARSurgUK Survey

Thank you for agreeing to take part in this brief survey as part of STARSurgUK.

The information you provide here will help us guide the content and structure of future STARSurgUK projects and will be essential for the ongoing growth and success of this world-first student network. We want to know about:

● Your previous experience of audit and research
● Any problems you experience registering the STARSurgUK audit
● Your perceptions of being involved in research and audit as a medical student

All data entered here will be held anonymously - the addition of your email address below will simply allow us to match your responses prior to and after having participated in the STARSurgUK audit and will not be passed onto any third party organisations.

The survey will take roughly 5-10 minutes to complete.

With Best Wishes and good luck participating in the project!
The STARSurgUK Steering Committee

*1. Email address (only for linking responses before and after your STARSurgUK audit participation):

Student Experience Survey (Pre)

Demographics

***2. What is your gender?**

- ○ Female
- ○ Male

***3. What is your age?**

[ ]

***4. Which year of medical school studies are you in?**

- ○ 1
- ○ 2
- ○ 3
- ○ 4
- ○ 5
- ○ 6
- ○ 7+

***5. Will you intercalate during your medical course?**

- ○ Already have
- ○ Have not, but intend to
- ○ Have not and don't intend to
- ○ Not sure

Other (please specify)

[ ]

***6. Additional degrees completed:**

- ☐ No additional degree
- ☐ BSc
- ☐ MSc
- ☐ MD
- ☐ PhD

Other (please specify)

[ ]

*7. Have you received NIHR Good Clinical Practice (GCP) certification?

- Yes
- No, but I know what GCP certification is
- No, and I don't know what GCP certification is

*8. What career pathway is currently of MOST interest to you?

- Anaesthesia
- Emergency Medicine
- General Practice
- Intensive Care Medicine
- Medicine (including all sub-specialties)
- Obstetrics and Gynaecology
- Occupational Medicine
- Ophthalmology
- Paediatrics
- Pathology
- Psychiatry
- Public Health
- Radiology
- Surgery (including all sub-specialties)

Other (please specify)

Prior Research/Audit Experience

The following questions help establish the baseline audit and research experience of STARSurgUK participants so that the right level of information and support is provided for you.

PRIOR TO HAVING BEEN INVOLVED IN THE STARSurgUK AUDIT...

*9. How many audits had you previously completed?

- 0
- 1
- 2
- 3
- 4
- 5+

*10. How many research projects (laboratory based) had you previously completed?

- 0
- 1
- 2
- 3
- 4
- 5+

*11. How many research projects (clinical) had you previously completed?

- 0
- 1
- 2
- 3
- 4
- 5+

***12. How many abstracts have you submitted to regional, national, or international POSTGRADUATE conferences previously (total accepted AND rejected)?**

- 0
- 1
- 2
- 3
- 4
- 5+

***13. How many poster presentations had you previously given?
(At regional, national, or international POSTGRADUATE conferences)**

- 0
- 1
- 2
- 3
- 4
- 5+

***14. How many oral presentations had you previously given?
(At regional, national, or international POSTGRADUATE conferences)**

- 0
- 1
- 2
- 3
- 4
- 5+

***15. How many peer-reviewed PubMed listed publications do you currently have?
(Original research and reviews only. Excludes letters, comment, opinion, editorials, technical tips etc)**

- 0
- 1
- 2
- 3
- 4
- 5+

***16. How many Research Ethics Committee (REC) or Institutional Review Board (IRB) applications (or their equivalent) had you previously submitted?**

- Not heard of these before
- Heard of, but not submitted any
- 1
- 2
- 3
- 4
- 5+

***17. Had you previously undertaken any work within a trainee collaborative research group previously?**
(e.g. http://www.asit.org/resources/collaboratives)?

- Yes
- NO, but AWARE that such collaboratives existed
- NO, and UNAWARE such collaboratives existed

Audit Registration

***18. Have you or your team ATTEMPTED to register the STARSurgUK 2013 audit at a UK centre?**

- Yes
- No

Audit Registration (2)

***19. Have you SUCCESSFULLY registered your audit at your chosen centre's audit department?**

- Yes
- No

Student Experience Survey (Pre)

Audit Approved

20. Please briefly detail any difficulties (if any) you faced in gaining audit approval at your centre:

Audit Not Approved

***21. Please state at which centre you unsuccesfully attempted to register your audit (Centre Name, Location):**

***22. Please detail why your audit was not approved/you were unable to gather support: (You may wish to copy and paste email content or add direct quotations from audit/R&D departments to furnish your answer. This will be important in aiding our understanding of the barriers faced by students in registering their STARSurgUK audits so we can address them in the next study)**

Audit Not Registered

*23. For what reason were you unable to register the STARSurgUK audit at your chosen centre? (Select all that apply)

- [ ] Your centre did not have an audit office
- [ ] Unable to contact the audit office at your centre
- [ ] Difficulty in completing the details required for the audit application form
- [ ] Unable to establish a mini-team with whom to complete the audit
- [ ] Unable to find a consultant willing to oversee the project
- [ ] The audit office would not approve this project as an audit
- [ ] The audit office required that you approached the research & development department at your centre prior to reviewing your audit form
- [ ] Other

(please specify)

Student Experience Survey (Pre)

Baseline Confidence Measures

***24. Prior to your involvement with STARSurgUK, how confident did you feel in the following?**

	Very Unconfident	Unconfident	Neutral	Confident	Very Confident
C. Writing an audit or research protocol	◌	◌	◌	◌	◌
B. Knowledge of the clinical audit cycle	◌	◌	◌	◌	◌
A. Distinguishing the differences between audit, service evaluation and research	◌	◌	◌	◌	◌
D. Approaching clinical staff to help you formulate an audit/research protocol	◌	◌	◌	◌	◌
E. Approaching clinical staff to form a team to help you complete an audit/research protocol	◌	◌	◌	◌	◌
H. How to collect data in the clinical setting	◌	◌	◌	◌	◌
G. How to contact your hospital's clinical audit department	◌	◌	◌	◌	◌
F. How to fill out an audit registration form	◌	◌	◌	◌	◌
I. How to present your results in a scientific manner	◌	◌	◌	◌	◌

Student Networks

*25. Please indicate your agreement with the following statements:

	Strongly Disagree	Disagree	Neutral	Agree	Strongly Agree
A. I have previously found it easy to participate in an audit/research project with students from other medical schools	○	○	○	○	○
B. I currently have ample opportunity to mix with surgically-minded students from other medical schools	○	○	○	○	○
C. I would value more inter-school networking opportunities with students interested in surgical careers	○	○	○	○	○

Student Experience Survey (Pre)

Academic Career Interest

***26. Please indicate your agreement with the following statements:**

	Strongly Disagree	Disagree	Neutral	Agree	Strongly Agree
B. I AM interested in applying for an academic foundation post	◯	◯	◯	◯	◯
C. I am NOT interested in pursuing an career in clinical academia	◯	◯	◯	◯	◯
A. I AM aware of the structure of academic training pathways in the UK	◯	◯	◯	◯	◯

Student Experience Survey (Pre)

Concluding Statements

***27. Please indicate your agreement with the following statements:**

	Strongly Disagree	Disagree	Neutral	Agree	Strongly Agree
A. Participation in clinical audit IS straightforward	○	○	○	○	○
B. Participation in audit IS important and relevant as a medical student	○	○	○	○	○
C. I would NOT be interested in participating in a registrar-led research collaborative project in the future	○	○	○	○	○
D. The collaborative model for publication is NOT fair for all participants	○	○	○	○	○

Student Experience Survey (Pre)

SURVEY COMPLETE

Thank you kindly for your input, your opinions are valued greatly.

Please keep an eye out for the post-audit questionnaire that will be distributed shortly following the date of completion of final follow-up.

Good luck with your audit!

Best Wishes,

The STARSurgUK Steering Committee

Email: STARSurgUK@gmail.com

Follow us on Twitter: @STARSurgUK
