# Supplementary material for: Promoting research and audit at medical school: evaluating the educational impact of participation in a student-led national collaborative study
Source: BMC Med Educ. 2015 Mar 13;15:47. doi: 10.1186/s12909-015-0326-1 (PMC4456723; doi:10.1186/s12909-015-0326-1)
Supplement: Additional file 2: — Post-questionnaire. [file 12909_2015_326_MOESM2_ESM.pdf]

Student Experience Survey (Part II - Post Audit)

Introduction to STARSurgUK Survey

Thank you for agreeing to take part in this brief follow-up survey as part of STARSurgUK.

Please take a moment to watch our DEBRIEF PRESENTATION:

http://www.youtube.com/watch?v=EYtS6M1Lw9s

prior to completing this questionnaire.

We want to know about:

- Your experiences and the lessons learnt from your involvement in STARSurgUK
- Changes in your perceptions of audit and research
- Areas of difficultly in implementing the audit protocol locally
- Areas for improvement for STARSurg's project in 2014

The information you provide here will help us guide the content and structure of STARSurg2014 and will be essential for the ongoing growth and success of this world-first student network - thank you.

All data entered here will be held anonymously. The survey will take roughly 5-10 minutes to complete.

The STARSurgUK Steering Committee

*1. Email address (for linking pre- and post-survey responses):

PLEASE ENTER THE SAME EMAIL ADDRESS YOU USED FOR THE PRE-STARSurgUK SURVEY

2. If you are unsure as to the email address you used for the pre-audit survey please enter any possible alternative email address(es) here.

Student Experience Survey (Part II - Post Audit)

Demographics

*3. Please indicate whether you read/watched/attended the following:

	Yes	No
STARSurgUK Protocol (pdf).	○	○
STARSurgUK Protocol Presentation (slideshare): http://www.slideshare.net/starsurguk/star-surg-uk-presentation	○	○
STARSurgUK Youtube Video: http://www.youtube.com/watch?v=_Eb3fjzbcTo	○	○
STARSurgUK National Meeting (RCS England), November 2013	○	○
STARSurgUK Weekly Twitter Live Forums, October - November 2013	○	○
STARSurgUK Post-Audit Presentation (youtube):	○	○

*4. Will you intercalate during your medical course?

- ○ Currently intercalating
- ○ Already have
- ○ Have not, but intend to
- ○ Have not and don't intend to
- ○ Not sure
- ○ Unable to intercalate at my university
- ○ Graduate entry course

Other (please specify)

[ ]

*5. Have you received NIHR Good Clinical Practice (GCP) certification?

- ○ Yes
- ○ No, but I know what GCP certification is
- ○ No, and I don't know what GCP certification is

*6. What career pathway is currently of MOST interest to you?

- Anaesthesia
- Emergency Medicine
- General Practice
- Intensive Care Medicine
- Medicine (including all sub-specialties)
- Obstetrics and Gynaecology
- Occupational Medicine
- Ophthalmology
- Paediatrics
- Pathology
- Psychiatry
- Public Health
- Radiology
- Surgery (including all sub-specialties)

Other (please specify)

STARSurgUK Completion

***7. Did you successfully complete the STARSurgUK audit in 2013 at a UK centre?**

- ○ Yes
- ○ No

STARSurgUK Successfully Completed

***8. Please state at which centre you successfully completed your audit (Centre Name, Location):**

***9. Which audit period did you complete:**

- Period 1
- Period 2
- Period 3
- Period 1 & 3

Other combination (please specify)

***10. At present, have you submitted your finalised datasets and authorship excel sheets to STARSurgUK?**

- Yes
- No

***11. STARSurgUK welcome collaborators to present data they have collected to their local departmental audit meeting.**

Have you or do you intend to present your findings to an audit meeting at your centre?

- Yes, have presented
- Yes, intend to present
- No

Student Experience Survey (Part II - Post Audit)

Audit Not Registered

***12. For what reason were you unable to complete the STARSurgUK audit at your chosen centre? (Select all that apply)**

- [ ] Your centre did not have an audit office
- [ ] Unable to contact the audit office at your centre
- [ ] Difficulty in completing the details required for the audit application form
- [ ] Unable to establish a mini-team with whom to complete the audit
- [ ] Unable to find a consultant willing to oversee the project
- [ ] A surgeon(s) at your centre refused to be included in the audit
- [ ] The audit office would not approve this project as an audit
- [ ] The audit office required that you approached the research & development department at your centre prior to reviewing your audit form
- [ ] Difficulty in obtaining the data points required by the STARSurgUK protocol
- [ ] Difficulty in gathering sets of notes
- [ ] Your mini-team disbanded
- [ ] You felt inadequately supported by your mini-team
- [ ] Other

(please specify)

***13. How could we have assisted you further in remaining involved in STARSurgUK to the point of completion? (Select all that apply)**

- [ ] Addition of a briefer, summative protocol
- [ ] A simpler audit topic
- [ ] Fewer data points for collection
- [ ] Better support from your local lead
- [ ] Better support from the steering committee

Other (please specify)

Post-Audit Confidence Measures

***14. After your involvement with STARSurgUK, how confident did you feel in the following AUDIT/RESEARCH domains?**

	Very Unconfident	Unconfident	Neutral	Confident	Very Confident
I. How to present your results in a scientific manner	○	○	○	○	○
E. Approaching clinical staff to form a team to help you complete an audit/research protocol	○	○	○	○	○
J. Understanding the role of the Research Ethics Committee (REC) and/or Institutional Review Board (IRB)	○	○	○	○	○
D. Approaching clinical staff to help you formulate an audit/research protocol	○	○	○	○	○
A. Distinguishing the differences between audit, service evaluation and research	○	○	○	○	○
B. Knowledge of the clinical audit cycle	○	○	○	○	○
H. How to collect data in the clinical setting	○	○	○	○	○
F. How to fill out an audit registration form	○	○	○	○	○
C. Writing an audit or research protocol	○	○	○	○	○
G. How to contact your hospital's clinical audit department	○	○	○	○	○

***15. After your involvement with STARSurgUK, how confident did you feel in the following CLINICAL domains?**

	Very Unconfident	Unconfident	Neutral	Confident	Very Confident
A. Perioperative management of the surgical patient	○	○	○	○	○
B. Types of operation	○	○	○	○	○
C. Indications for different operations	○	○	○	○	○
D. Identifying post-operative complications	○	○	○	○	○

Student & Post-Graduate Networks

*16. Please indicate your agreement with the following statements:

	Strongly Disagree	Disagree	Neutral	Agree	Strongly Agree
C. I would value more inter-school networking opportunities with students interested in surgical careers	◯	◯	◯	◯	◯
B. STARSurgUK did NOT provide opportunity to mix with surgically-minded students from other medical schools	◯	◯	◯	◯	◯
E. Having participated in STARSurgUK I AM more likely to engage with post-graduate research collaboratives in surgery	◯	◯	◯	◯	◯
F. I DID feel part of the STARSurgUK team whilst completing the 2013 audit	◯	◯	◯	◯	◯
D. I am NOT aware of the existence of post-graduate research collaboratives in surgery	◯	◯	◯	◯	◯
A. STARSurgUK HAS made it easier to participate in an audit/research project with students from other medical schools	◯	◯	◯	◯	◯

17. Did you attend the National Research Collaborative meeting (#NRCM2013) at RCS (England) on the 6th December 2013? http://www.nationalresearch.org.uk/

◯ Yes, attended in person

◯ Yes, as a virtual delegate

◯ No, but I was aware it was happening

◯ No, and I was UNaware it was happening

Student Experience Survey (Part II - Post Audit)

Academic Career Interest

***18. Please indicate your agreement with the following statements:**

	Strongly Disagree	Disagree	Neutral	Agree	Strongly Agree
B. I AM interested in applying for an academic foundation post	○	○	○	○	○
C. I am NOT interested in pursuing an career in clinical academia	○	○	○	○	○
A. I AM aware of the structure of academic training pathways in the UK	○	○	○	○	○

Student Experience Survey (Part II - Post Audit)

Concluding Statements

*19. Please indicate your agreement with the following statements:

	Strongly Disagree	Disagree	Neutral	Agree	Strongly Agree
D. The collaborative model for publication is NOT fair for all participants	◯	◯	◯	◯	◯
B. Participation in audit IS important and relevant as a medical student	◯	◯	◯	◯	◯
C. I would NOT be interested in participating in a registrar-led research collaborative project in the future	◯	◯	◯	◯	◯
A. Participation in clinical audit IS straightforward	◯	◯	◯	◯	◯

Student Experience Survey (Part II - Post Audit)

Logistics

*20. Did you have a 'local lead' for your area?

- ○ Yes
- ○ No

*21. Please indicate your agreement with the following statements:

	Strongly Disagree	Disagree	Neutral	Agree	Strongly Agree	Not Applicable
C. Queries raised to the STARSurgUK committee were answered adequately	○	○	○	○	○	○
D. Queries raised to the STARSurgUK committee were answered in a timely fashion	○	○	○	○	○	○
B. I felt overwhelmed by the VOLUME of information sent to me via email	○	○	○	○	○	○
A. The NUMBER of email updates distributed was sufficient	○	○	○	○	○	○
E. I felt supported by my local lead	○	○	○	○	○	○

*22. Please indicate THREE areas from STARSurgUK's 2013 audit in which you would have appreciated further support:

- ☐ Understanding of the generic audit process
- ☐ Understanding of the STARSurgUK audit protocol & data points
- ☐ Approaching a supervising consultant
- ☐ Establishing a local mini-team
- ☐ Registering your audit at your local centre
- ☐ Identifying elligible patients
- ☐ Inpatient follow-up
- ☐ Retrieving patient notes
- ☐ Completing the STARSurgUK excel sheet
- ☐ Submitting the STARSurgUK data sheet via an NHS.net/.org email address
- ☐ Presenting your audit findings to a local departmental meeting

Other (please specify)

[ ]

2014 and onwards...

***23. Would you be interested in participating in STARSurg's 2014 project, which will see the collaborative expand across Europe?**

- [ ] Yes
- [ ] No

if no (please explain your reasons)

***24. Please in which month(s) you feel it would be most suitable to run STARSurg in 2014 (please select all that apply):**

- [ ] January
- [ ] February
- [ ] March
- [ ] April
- [ ] May
- [ ] June
- [ ] July
- [ ] August
- [ ] September
- [ ] October
- [ ] November
- [ ] December

***25. Please indicate the maximum length in which you are likely to be in the same hospital on clinical placement during 2014-15?**

- ( ) <1 week
- ( ) 1-2 weeks
- ( ) 3-4 weeks
- ( ) 5-6 weeks
- ( ) 7-8 weeks
- ( ) 8+ weeks

***26. We intend to hold another STARSurgUK meeting in 2014.**

Please indicate how interested you would be in the following sessions:

	Very Uninterested	Uninterested	Neutral	Interested	Very Interested
Formal Good Clinical Practice (GCP) training	◯	◯	◯	◯	◯
Workshop: Designing a randomised control trial	◯	◯	◯	◯	◯
Lecture: How to deliver a research presentation	◯	◯	◯	◯	◯
Lecture: How to get published	◯	◯	◯	◯	◯
Workshop: Critically appraising a paper	◯	◯	◯	◯	◯
Lecture: How to write an abstract	◯	◯	◯	◯	◯
Workshop: Writing a research protocol	◯	◯	◯	◯	◯
Lecture: How to design a research poster	◯	◯	◯	◯	◯

Do have any other suggestions of sessions of potential interest (please state here):

Student Experience Survey (Part II - Post Audit)

SURVEY COMPLETE

Thank you kindly for your ongoing input, your opinions are valued greatly.

We hope you have enjoyed being part of STARSurgUK's inaugural 2013 audit and we very much hope you will continue to be involved with our exciting pan-European plans for 2014.

Please follow us on @STARSurgUK and like us on facebook.com/STARSurgUK for regular updates and details of upcoming opportunities.

Post-analysis, we keep you informed of STARSurgUK's finding and plans for presentation and publication, with which you will ALL be citable under the 'STARSurgUK' collaborative banner.

Best Wishes,

The STARSurgUK Steering Committee

Email: STARSurgUK@gmail.com, aneel.bhangu@nhs.net
